# State Medical Society

**Published:** 1858-06

**Authors:** 


### STATE MEDICAL SOCIETY.

Not being able to attend the meeting of our State Medical Society at Mt. Pleasant, we clip from the *Home Journal*, whose editors, Messrs. ELLIOTT & EDWARDS, have so kindly furnished.

The annual meeting of the Iowa State Medical Society was held in this city on the 9th and 10th inst. Owing to the incessant rains, and impassable roads, the delegations from the interior were prevented from attending, and many of the Physicians of Henry county were unable to reach the city on account of the high waters, &c.

The profession was represented by delegates from Dubuque, Davenport, Iowa City, Keokuk, Burlington, Fairfield, Salem, and Leclaire, together with a full representation from our own City.

The society was called to order by the President, Dr. THOMAS SIVETER, of Henry county, and in the absence of the permanent Secretary, Dr. A. W. McClure was elected *pro tem.*

After the transaction of the usual preliminary business, Dr. McGugin, of Keokuk, suggested that more time should be spent in discussion of Medical subjects for the mutual improvement of members of the profession. He wished to hear reports of cases, and the treatment of those cases, suggesting improvements where a difference of opinion existed, &c. He said he had been a student all his life, and was still most anxious to learn; and had no doubt there was many things yet to learn.

Dr. Berkey remarked that he merely wished to ask a question. Why is it that gentlemen of Dr. McGugin's age, who have been hard students for many years, are not through with their medical studies, when we see scores of *young men* who are "*thorough perfect masters*" of the science of medicine? (Dr. B. no doubt intended by this question, propounded ironically, to remind the young disciples of Æsculapius that there was *something* yet for them to learn.)

The President, Dr. Siveter, read his annual message, a very interesting document which we regret has not been furnished us for publication. An election for officers was held on the afternoon of the 9th, when Dr. JOHN H. RAUCH, of Burlington, was chosen President. Dr. E. S. BARROWS, of Davenport, Vice President, and Dr. J. W. H. BAKER, Secretary.

The usual committees were appointed and delegates elected to the National Medical Association, to meet at Louisville, Ky., in May 1859.

LECTURE ON QUACKERY.

Dr. McGugin delivered a lecture on Wednesday evening in the Universalist Church. Owing to the hydropathic character of the weather there was not a full house. The Doctor is a man of fine abilities, and an agreeable speaker. Many of his positions were entirely *original*, and somewhat startling. He claimed that medicine was an *inspired* science, inaugurated under the administration of Moses; and remaining *intact* until the present time. The Speaker spoke in terms of severity of the army of *quacks* that are inundating the country, endangering life and health; pretending to heal diseases of which they are grossly ignorant; administering nostrums of which they know less than nothing; and all for the purpose, not of curing the sick, but to rob the people and feed upon their money. The exalted character of the *regular* profession was dwelt on at some length, and if the Doctor failed to prove *their* high station from the *inspired* portion of the scriptures, he found ample "documents" in the *Apocrypha* for his purpose. We confess our bowels of compassion yearned over a few "irregulars" who were present, and on whose devoted heads the vials of professional wrath were so freely poured out. We hope it may prove a "savor of life" unto many of them, and also to their *patients*.

At the conclusion of the lecture the Medical Faculty, and a number of ladies and citizens repaired to the

BANQUET AT THE BRAZELTON HOUSE,

Prepared under the direction of the Henry County Medical Society, for the entertainment of their guests. At ten o'clock the company set down to a sumptuous repast got up by the proprietors with lavish expenditure and excellent *taste*. His Excellency Governor Lowe occupied the head of the table, and Dr. L. P. Hamline presided at the banquet.

The Mount Pleasant Brass Band enlivened the exercises of the dining-room with

"Such sweet, such melting strains!
Their soft harmonious cadence rises now,
And swells in solemn grandeur to its height!
Now sinks to mellow notes—now dies away—
But leaves its thrilling memory on my ear."

After a perfect *diagnosis* of the supper was taken by all the practitioners, the cloth was removed, and Dr. Stebbins read the following

REGULAR TOASTS.

*Iowa and her Governor:* Of the first it may be said her resources are inexhaustible, and, like the powers of the human mind, may be increased and developed to an indefinite extent by cultivation. To the temperate and industrious, she offers a home and a field worthy of their noblest efforts. Of the latter, we may add he is worthy to be the executive of such a Commonwealth, and as one evidence of this we will point to the fact that he went through an animated political canvass and was elected Governor without incurring one unkind or disparaging word from his political opponents.

Gov. Lowe responded in a brief and pertinent address, setting forth a few of the leading characteristics in the *greatness* of Iowa, suggesting what he deemed essential in her policy to ensure future growth, and closed by an expression of thanks to the members of the medical profession who so kindly and flatteringly alluded to himself. The remarks of the Governor were listened to with great attention and warmly applauded.

*The Medical Profession*, Pioneers and faithful laborers in the mines of knowledge, they have already collected from every conceivable source an amount of precious ore sufficient to warrant the hope that they will ultimately build up such a fabric of science as will be the crowning glory of intellect and genius, which will insure the restoration of all curable cases of disease, and greatly mitigate the sufferings of the hopeless.

Dr. Cochran, of Iowa City, replied to this sentiment in an eloquent and graphic review of the progress which the science of Medicine had made since the days of Hippocrates, and also referred to statistics of mortality showing the great decrease in fatal cases as medical science is developed. Dr. Cochran is a young man of fine talents, and bids fair to occupy a leading position in his profession. We expect to hear of his brilliant future among the M. D.'s of Iowa.

*The State Medical Society*, Though young in years and few in numbers, may its proceedings be such as to inspire the zeal, energy, and hearty co-operation of the profession, until it shall be regarded by all as an honor to Iowa and an important aid to the existing sources of Medical Science, and may its early founders live to rejoice in its good fruits.

Dr. Rauch, President of the society, responded to this toast in his happy manner, pledging his efforts to increase the strength and usefulness of the association, and earnestly requesting hearty co-operation from his associates in the noble science of medicine. Dr. R. is also a young man of superior talents,—an indefatigable worker in the practice and science of physic, and is fast attaining a prominence in his profession which talent and industry combined are sure to win.

*Our Guests*, The members of the Iowa State Medical Society, We bid them welcome to Mt. Pleasant and to the festive board, for we appreciate the worthy and honorable motives that brought them here, and we assure them that we shall ever cherish the most grateful recollections of our meeting and the kindest wishes, both for their individual success as physicians, and their general prosperity and welfare.

Dr. Barrows, of Davenport, responded in behalf of the members of the Society present; and we regret that our absence from the room at the time prevented us from making note of his remarks.

*The Medical Department of Iowa University*, This institution merits the confidence of the profession because of the capacity, energy and scientific attainments of its Professors. We trust that it has passed the ordeal of infancy, and that it may prove in the future what it has been in the past, an instrument for the elevatien of the standard of medical education in the State and a beacon light of medical science for the great west.

Dr. McGugin, Professor of the Medical Department of the Iowa State University, replied to the sentiment at some length, in a sketch of the rise, progress, and present importance of the institution—spoke hopefully of the future renown of her students already sent forth to battle disease and death in the community; and paid a very glowing tribute to the spirit of progress manifested in all ranks of society in this great State.

*The Press*, The promoter and preserver of science, and the means by which all knowledge is diffused among men: may it ever be pure as virtue itself, and while pure, free as the winds of Heaven.

D. S. Elliott, of the *Home Journal*, responded in a short speech, showing a striking analogy between the medical and editorial professions, and concluded with a sentiment to the Ladies present.

VOLUNTEER TOASTS.

By Dr. J. C. Stone—

*To the Members of the Henry County Medical Society*—Your guests would embrace this opportunity to acknowledge your genial hospitality, bounteous entertainment, and would commend your generosity to the medical profession.

By Dr. W. S. Marsh—

*Mr. President and Gentlemen, members of a high, noble, and time honorable profession*, May it be your ambition to excel in every branch of the science of medicine. May you ever be distinguished by intelligence, dignity, courtesy and integrity from the quackeries of the present age. May your lives be useful, happy and full of years, and your names remembered long after you have passed away as benefactors of your race.

By Dr. S. Stebbins—

*The Ladies: our dearest companions in the labors, the joys and sorrows of life,* We welcome them to the medical profession as our equals, and ardently hope they may prove themselves as much our superiors in prescribing for, as they ever have been in nursing the sick and afflicted.

The company separated about the wee sma' hours of the night, mutually gratified with their social intercourse. We hope our medical friends from abroad enjoyed their visit to our city, and we have only to regret that unfavorable weather prevented so many from partaking of the hospitality of Mount Pleasant.

